# Acclimatization of the Crustose Coralline Alga *Porolithon onkodes* to Variable pCO_2_

**DOI:** 10.1371/journal.pone.0087678

**Published:** 2014-02-05

**Authors:** Maggie D. Johnson, Vincent W. Moriarty, Robert C. Carpenter

**Affiliations:** Department of Biology, California State University, Northridge, California, United States of America; University of New South Wales, Australia

## Abstract

Ocean acidification (OA) has important implications for the persistence of coral reef ecosystems, due to potentially negative effects on biomineralization. Many coral reefs are dynamic with respect to carbonate chemistry, and experience fluctuations in pCO_2_ that exceed OA projections for the near future. To understand the influence of dynamic pCO_2_ on an important reef calcifier, we tested the response of the crustose coralline alga *Porolithon onkodes* to oscillating pCO_2_. Individuals were exposed to ambient (400 µatm), high (660 µatm), or variable pCO_2_ (oscillating between 400/660 µatm) treatments for 14 days. To explore the potential for coralline acclimatization, we collected individuals from low and high pCO_2_ variability sites (upstream and downstream respectively) on a back reef characterized by unidirectional water flow in Moorea, French Polynesia. We quantified the effects of treatment on algal calcification by measuring the change in buoyant weight, and on algal metabolism by conducting sealed incubations to measure rates of photosynthesis and respiration. Net photosynthesis was higher in the ambient treatment than the variable treatment, regardless of habitat origin, and there was no effect on respiration or gross photosynthesis. Exposure to high pCO_2_ decreased *P. onkodes* calcification by >70%, regardless of the original habitat. In the variable treatment, corallines from the high variability habitat calcified 42% more than corallines from the low variability habitat. The significance of the original habitat for the coralline calcification response to variable, high pCO_2_ indicates that individuals existing in dynamic pCO_2_ habitats may be acclimatized to OA within the scope of *in situ* variability. These results highlight the importance of accounting for natural pCO_2_ variability in OA manipulations, and provide insight into the potential for plasticity in habitat and species-specific responses to changing ocean chemistry.

## Introduction

Anthropogenic CO_2_ emissions have been rising steadily since the Industrial Revolution with the increase in fossil fuel consumption, agriculture, and land development [1], resulting in greater concentrations of CO_2_ entering the surface ocean [2]. Ocean acidification (OA) refers to the equilibration of atmospheric CO_2_ with the surface ocean, and the subsequent changes to seawater carbonate chemistry. These reactions are manifest as a decrease in mean ocean pH, carbonate ion concentration (CO_3_^2−^) and CaCO_3_ saturation state (Ω), an increase in pCO_2_ and bicarbonate ion concentration (HCO_3_^−^), and no change in total alkalinity (A_T_). Saturation state is an important factor that influences rates of biomineralization, where CaCO_3_ precipitation is favored when Ω is >1 [3]. Changes in Ω associated with ocean acidification have important implications for biogenic calcifiers and for ecosystems that are structurally dependent on carbonate platforms, such as coral reefs [3]. The changes in carbonate chemistry due to OA have raised widespread concern, particularly because they are occurring at a rate that is at least 2 orders of magnitude faster than during previous glacial periods [4]. The accelerated rate of OA may outpace the ability of marine organisms to adapt to the changing environment [5].

Near future projections for atmospheric CO_2_ gas concentrations, such as in the Representative Concentration Pathways (RCPs) [6], are based on open ocean conditions where carbonate chemistry varies predictably over time and mean ocean pH is reported to be 8.02–8.07 [7]. Seawater chemistry in near shore coastal ecosystems, such as upwelling regions [8], [9], coral reefs [10]–[12], estuarine systems, kelp forests [7] and natural CO_2_ vents [13]–[16], is both spatially and temporally dynamic within a habitat [17]. Swings in diel pH frequently exceed the forecasted projections of ocean acidification for the end of the century [7], [12], [18]–[20]. The recent development of autonomous sensors that record high frequency *in situ* pH data over time [21], [22] have shed light on the extent of pH variability within habitats and across ecosystems [7], [12].

In coral reef ecosystems, particularly shallow reef flats, carbonate chemistry varies over the course of a day [17], with tidal cycles [23], [24], and even between upstream and downstream habitats [25], [26]. The shallow reef benthos often is dominated by biogenic calcifiers that can create high pCO_2_ and low pH conditions by depleting total alkalinity (A_T_) [27] and releasing CO_2_ during the process of calcification [28], [29]. The interplay of reef metabolism and carbonate chemistry thus creates a feedback that influences the magnitude of pH variability that resident organisms experience [12], [30]–[32]. A feedback also exists in the balance of photosynthesis and respiration, with peaks and lows in photosynthesis during the day and night respectively [10], [33], [34]. Exposure of resident marine organisms to dynamic swings in diel pH may influence the plasticity of organismal responses to changing carbonate chemistry, yet variation in pH has been incorporated in only a few ocean acidification manipulations [17], [35]–[37].

To date, much of the ocean acidification literature has focused on the effects of decreased pH/increased pCO_2_ on biological responses of marine organisms in static laboratory mesocosm experiments by maintaining steady CO_2_ treatments over time [38]. Although the magnitude of responses varies, the overall effect of ocean acidification on marine calcifiers primarily has been negative [38]–[40]. These studies have been critical to advancing the understanding of CO_2_ effects on marine organisms, but may underestimate the importance of natural fluctuations in pCO_2_ on organismal responses. For instance, coral recruits exposed to ecologically relevant oscillations between ambient and high pCO_2_ experienced enhanced growth and survival when compared to recruits exposed to ambient or high pCO_2_ conditions alone [36]. By understanding the role that natural variability plays towards influencing species responses to changing carbonate chemistry we can gain insight into the potential for organisms to acclimatize and adapt to ocean acidification. This issue is of primary concern because the accelerated rate of ocean acidification is unprecedented over geological history [41] and may preclude the adaptive potential of some marine species. Little is known about species acclimatization potential to ocean acidification, and studies are beginning to explore the concept of acclimation in the context of ocean acidification [42]. Understanding how organisms already may be acclimatized in a rapidly changing environment can improve our understanding of how coral reef community structure may change in the near future. Marine ecosystems in which organisms are exposed frequently and consistently to environmental fluctuations, such as shallow coral reef flats [7], [10], [12], provide an *in situ* platform in which to explore the potential for acclimatization to ocean acidification.

Although much of reef net CaCO_3_ deposition occurs through biogenic calcification by scleractinian corals [43], another important group of reef builders are crustose coralline algae (CCA) [44], [45]. Crustose corallines serve many important ecological functions by contributing to reef primary productivity [46]–[48], facilitating settlement of coral larvae [49]–[51], and maintaining the structural integrity of the coral reef framework by cementing reef fragments together [52], [53]. Corallines precipitate the most soluble polymorph of CaCO_3_, high-magnesium (Mg) calcite [54], and have been shown to be sensitive to ocean acidification. Exposure to high pCO_2_ conditions reduces coralline calcification and growth [40], [55]–[60], and inhibits the function of the chemical cue produced by corallines and/or their microbial assemblages that facilitates coral settlement [61]–[64]. Despite their important role in coral reef ecosystems relatively few studies have addressed the effects of OA on tropical coralline algae. Furthermore, studies have yet to explore the effects of variable pCO_2_ on crustose corallines, or their potential for acclimitization. *Porolithon onkodes* (synonymous with *Hydrolithon onkodes* [65]) is an important reef-building coralline alga that is abundant throughout the Indo-Pacific in shallow reef systems and has been shown to promote settlement in coral larvae [49], [51]. By exploring the response of *P. onkodes* from a naturally dynamic shallow reef flat to oscillating pH, we may better understand the adaptability of an important reef-building species to ocean acidification.

The aims of this study were, 1) to determine the effect of oscillating pCO_2_ on crustose coralline calcification, photosynthesis, and respiration, and 2) to determine the role of prior exposure to *in situ* pCO_2_ variability in influencing coralline responses to ocean acidification. To accomplish the first goal, we conducted tightly-controlled laboratory mesocosm experiments in Moorea, French Polynesia, in which we exposed *P. onkodes* to ambient, high, and oscillating pCO_2_ at levels ranging from ∼400 µatm to 660 µatm, the natural range in pCO_2_ on the back reef of Moorea. We hypothesized that exposure to high pCO_2_ alone would decrease *P. onkodes* calcification, as shown in previous studies, and that exposure to pCO_2_ oscillating within an ecologically relevant range would modulate the negative effect of high pCO_2_ on calcification. Further, we hypothesized that exposure to high pCO_2_ would enhance net photosynthesis in all corallines due to the potential promoting effects of increased dissolved CO_2_ on carbon fixation. To explore the potential for acclimatization to ocean acidification conditions, we used *P. onkodes* individuals collected from either low pH variability sites (reef crest, upstream) or high pH variability sites (200 m shoreward of the reef crest, downstream) within the same reef habitat. We hypothesized that individuals from downstream sites would calcify more in the variable pCO_2_ treatment than those from upstream sites because they were acclimatized *in situ* to variable pCO_2_. To our knowledge, this study is the first to quantify the response of coralline algae to variable pCO_2_ and to explore the potential for acclimatization of an important reef builder to high pCO_2_.

## Materials and Methods

### Site Description

This study was conducted during May and June 2010 in Moorea, French Polynesia as part of the Moorea Coral Reef Long-Term Ecological Research site (MCR LTER) under a Protocole D'accueil (Scientific Research Permit) to RCC from the Delegation a la Recherche de la Polynesie Français (unnumbered). Moorea is located 20 km west of Tahiti and is surrounded by a barrier reef that encloses a lagoon. Water circulation is driven by oceanic and wind-driven swells that break on the reef crest and force mostly unidirectional flow of water across the back reef and into the lagoon where it then exits through one of the reef passes [66]. In the back reef on the north shore of Moorea, water residence time is dependent on offshore wave height. The transit time for water flowing over the reef crest to habitats 200 m downstream is approximately 30–45 minutes [66] and is dependent on flow speed. The carbonate chemistry of water flowing across the back reef is altered by reef metabolism, with photosynthesis and calcification decreasing pCO_2_ over the reef during the day, and respiration and dissolution increasing pCO_2_ over the reef during the night [29]. The farther a parcel of water travels from the source of oceanic inflow over the reef crest, the longer the residence time and exposure to reef metabolism and the greater potential for change in the carbonate chemistry. As a result, reef organisms inhabiting upstream locations (*e.g*., fore reef and reef crest) are exposed to relatively constant pCO_2_ of incoming oceanic seawater, while the pCO_2_ experienced by organisms living in downstream habitats varies on a diel basis and with the environmental conditions of light and water flow that modulate reef metabolic processes and determine water residence time over the reef [25], [26], [31], [67], [68].

### Sample Collection

Samples of the common crustose coralline alga, *Porolithon onkodes*, were collected from upstream habitats on the shallow fore reef at a depth of 2–3 m on the north shore reef between Cook's and Opunohu Bays. Samples of the same species also were collected from the back reef approximately 200 m downstream of the reef crest at a similar depth. These two locations experience approximately the same flow [66], nutrient, and sedimentation [69] conditions. Individuals collected from the upstream location represent organisms exposed to a history of low pH variability, and individuals from the downstream location a history of higher pH variability [26], [31]. Crustose coralline samples were collected using a diamond-grit hole saw (40 mm outer diameter, 36 mm inner diameter) attached to a pneumatic drill. Coralline algal thalli were separated from the underlying dead coral skeleton to yield approximately 0.5 cm thick cores of *P. onkodes*. Coralline algal samples were returned to the lab and carefully cleaned of epibionts. The bottoms of the algal disks were covered with marine epoxy (Z-spar) to prevent exposure of the underlying carbonate to potential dissolution in treatment conditions. Samples were kept in a water table with a high exchange of fresh seawater, under ambient light, for three days to allow for healing. Only healthy appearing samples without obvious tissue damage were used in the experiment.

After samples were collected, the taxonomic identification of *Porolithon onkodes* was assigned based on Adey et al. (1982) [70] and Payri et al. (2000) [71]. We used gross morphological, reproductive, and trichocyte characteristics to identify each sample using simple laboratory procedures. Specimens were dried at 60°C for 48 hours, fragmented with diagonal cutters, and internal characteristics of the fragmented edges, including conceptacle size and shape, trichocyte arrangement, cellular organization, and margin morphology were examined under a dissecting scope. Identification of samples was verified by comparison to samples (N = 3) that had been previously confirmed by Dr. Robert Steneck (personal comm.).

### CO_2_ Enrichment Treatments

The CO_2_ enrichment treatments were established in a mesocosm facility at the Richard B. Gump South Pacific Research Station consisting of 6, 150-L tanks, each with independent lighting (250 W metal halide fixtures) and temperature control. Mesocosm tanks were operated in an open configuration with each tank receiving fresh, unfiltered seawater pumped from Cook's Bay at a rate of 140 ml min^−1^ tank^−1^ resulting in approximately two complete turnovers of water each day. Each tank was fitted with a clear plexiglass lid to reduce atmospheric gas exchange and stabilize treatment pCO_2_ levels. Light levels (PAR) were adjusted to maintain the light level in each tank at ∼600 µmol quanta m^−2^ s^−1^ (on a 12∶12 h light:dark cycle) that resulted in a daily integrated 12-h PAR similar to that measured at the collection sites (∼26 mol quanta m^−2^ d^−1^). The temperature control for each tank was set to a target value of 28.5°C which represents the ambient temperature on the reef during the austral winter (May/June).

To simulate the effect of atmospheric CO_2_ on ocean surface waters, treatment tanks were bubbled with either ambient air or CO_2_-enriched air to establish targeted pCO_2_ levels. CO_2_-enriched air was created using a solenoid-controlled gas regulation system (Model A352, Qubit Systems) that mixed pure CO_2_ with ambient air to achieve the desired partial pressure (sensu Edmunds *et al*. (2011)) [72]. The targeted pCO_2_ value for the high pCO_2_ treatment was 650 µatm, which represents the pCO_2_ at downstream sites just before dawn and the projected atmospheric pCO_2_ in 50 years based on carbon emission scenarios [6]. The three pCO_2_ treatment levels used in the experiment were ambient (∼400 µatm), high pCO_2_ (∼660 µatm), and oscillating pCO_2_ (samples were exposed to a treatment that alternated between 400/660 µatm).

The physical parameters of each tank were monitored throughout the experiment including light, salinity, temperature, and carbonate chemistry. Temperature was measured daily in the morning, afternoon, and evening using a digital thermometer with an accuracy of 0.05°C (Traceable Digital Thermometer, Thermo Fisher Scientific). Light was measured daily beneath the surface of the water and at the sample locations (∼15 cm above the bottom of the tank) in each tank using a 2π LiCOR quantum sensor connected to a LiCOR 1400 meter. Salinity was measured daily on water samples from each tank using a desktop YSI 3100 conductivity meter, and pH (total scale) was measured daily using a spectrophotometric technique (described below).

### Seawater Chemistry

Water samples were collected daily at 0900 hours in 20 mL glass scintillation vials for spectrophotometric analysis of pH (total scale). pH measurements were made immediately following collection using a spectrophotometer (Shimadzu UV-2450) and the indicator dye m-cresol purple according to standard operating procedure (SOP) 6 [73]. The temperature of the sample was controlled with a constant-temperature cell holder (Shimadzu) that maintained cell temperature at 25°C. The accuracy of the spectrophotometric technique was assessed by conducting pH measurements on certified reference material with a known pH (Tris Buffer in synthetic seawater, A. Dickson, Scripps Institution of Oceanography), and the mean accuracy of this technique was ±0.09% (N = 16). Water samples were collected every two days at 0900 hours for determinations of total alkalinity (A_T_) by siphoning treatment water into 250-ml borosilicate sample bottles [73]. Water samples for A_T_ were analyzed within 6 hours of collection after the water sample had equilibrated with laboratory room temperature. Total alkalinity (A_T_) determinations were made using a Mettler T50 automated titrator to conduct modified, open cell potentiometric titrations [74] according to standard operating procedure (SOP) 3b [73]. Titrations were conducted on 100-ml samples at a temperature of 24°C (samples were equilibrated with room temperature) with a DG115-SC pH probe (Mettler Toledo) calibrated using a three-point calibration with NBS buffers (pH 4.00, 7.00, 10.00). The mean accuracy of the AT determinations was ±0.5% (N = 6) compared to Certified Reference Materials (Reference Material for Oceanic CO2 Measurements, Batch 99, A. Dickson, Scripps Institution of Oceanography).

Water samples were collected at each site over five days in order to characterize the carbonate chemistry at the upstream and downstream sites. Integrated water samples were collected during the day (from 0800 to 1800) and during the night (from 1900 to 0400) by continuously sampling water (4 mL min^−1^) with a submersible autonomous water sampler. Upstream and downstream water samples were collected simultaneously. Water temperature was recorded at the time of sample collection and samples were returned to the lab and analyzed as described above within 24 h of collection. The results from all samples were processed in Microsoft Excel [74], and A_T_, pH, salinity and temperature were used to determine the remaining carbonate parameters using CO_2_SYS [75] with the dissociation constants of Mehrbach *et al*. (1973) [76] refit by Dickson & Millero (1987) [77].

**Figure 1:**
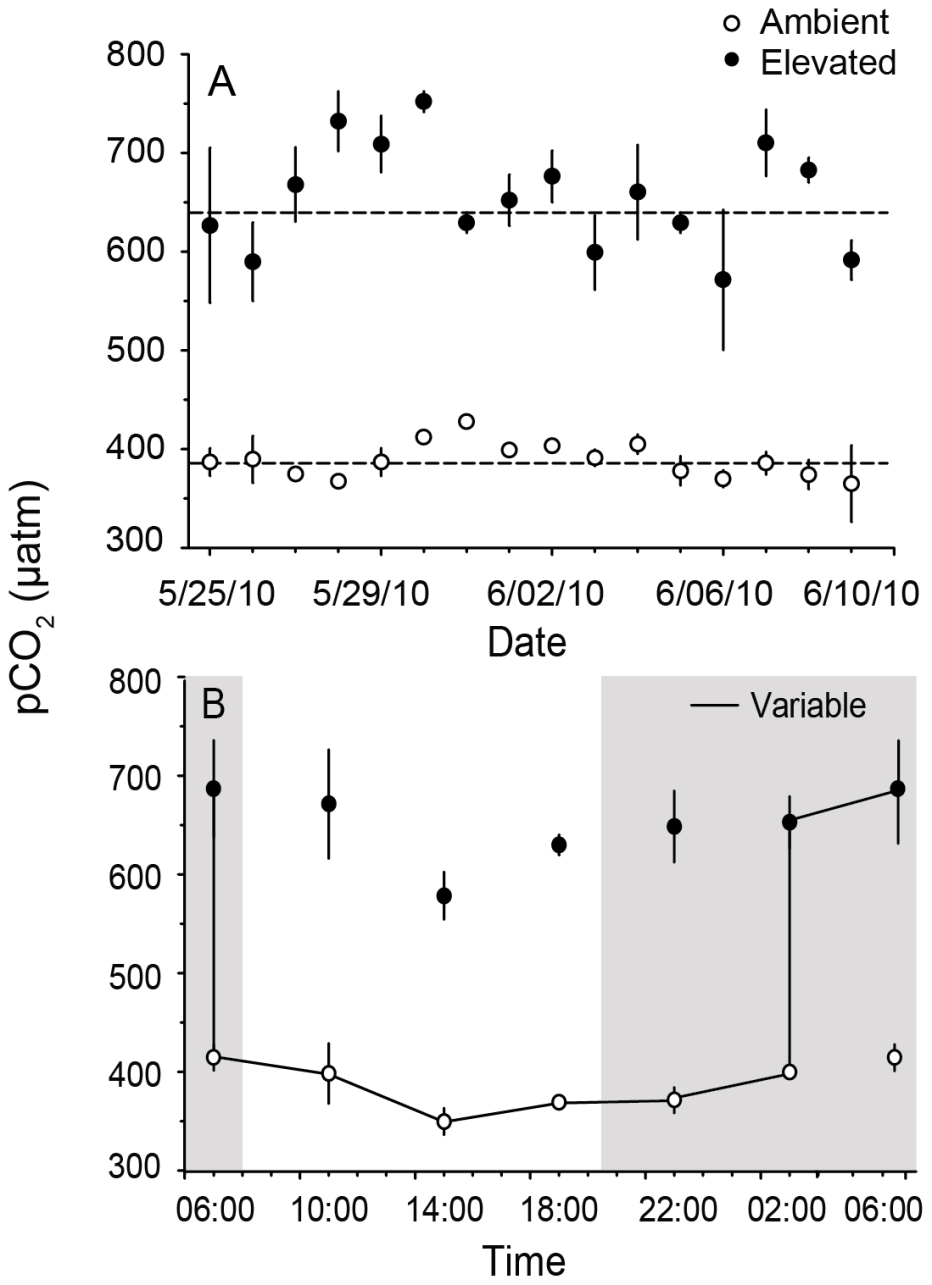
Mean daily and hourly pCO_2_ of treatment conditions. Mean (± SE) daily pCO_2_ of ambient (open circles) treatment tanks (N = 3), elevated (closed circles) treatment tanks (N = 3), and the overall treatment means for the duration of the experiment (dashed line) (A). Mean pCO_2_ of ambient and elevated treatments measured over one diel cycle (B). The solid line shows the transition of the variable treatment samples from ambient pCO_2_ into the elevated pCO_2_ treatment 0000 and then back into the ambient treatment at 0600. pCO_2_ was calculated using CO_2_SYS and measurements of total alkalinity and pH (total scale).

The environmental conditions in the established treatments were stable over the 14-d experiment. Temperature, salinity, light, and total alkalinity were similar across all treatments and also similar to ambient levels in the field (Table 1). The pCO_2_ treatments created by bubbling ambient and CO_2_-enriched air resulted in distinct, non-overlapping pH and pCO_2_ levels (Figure 1A) and saturation states of calcite (Ω_calc_) (Table 1). The pCO_2_ levels in the elevated treatments (680±15, mean ± SE) were 65% higher than in the ambient treatments (411±14) (Figure 1A) and Ω_calc_ was reduced by 28% from 5.67±0.04 to 4.09±0.06 as a result of a reduction in the concentration of carbonate. There was no temporal variation in pCO_2_ within the treatment tanks over the 2, 24-h periods that were sampled (Figure 1B).

**Table 1:** Carbonate chemistry measurements.

Treatment	Temp (°C)	Salinity (psu)	Light†	pH‡	TA (µmol/kg)	pCO_2_ (µatm)	Ω_Ca_	Ω_Ar_
Ambient	28.7±0.02	35.5±0.02	607±15	8.041±0.007	2311±8	417±16	5.58±0.13	3.72±0.09
Ambient	28.5±0.02	35.5±0.03	631±15	8.035±0.005	2312±7	404±21	5.66±0.15	3.78±0.10
Elevated	28.5±0.02	35.5±0.04	651±17	7.863±0.014	2315±8	667±30	4.12±0.15	2.75±0.10
Elevated	28.7±0.02	35.5±0.03	654±15	7.863±0.013	2314±3	663±42	4.17±0.19	2.78±0.12
Var/Amb	28.7±0.02	35.5±0.02	668±16	8.027±0.005	2329±6	432±6	5.52±0.06	3.68±0.04
Var/Elev	28.4±0.02	35.5±0.02	634±12	7.865±0.014	2322±3	666±67	4.17±0.32	2.78±0.21
								

Values (means ± SE) for physical variables in each tank over the course of the 14-d experiment. N = 14 sampling days for temperature, salinity, light, and pH, and N = 5 sampling days for TA, pCO_2_, Ω_Ca_, and Ω_Ar_.

† Light  =  photosynthetically active radiation (PAR, µmol quanta),

‡ pH  =  total scale pH units, Ω_Ca_  =  the saturation state of calcite, Ω_Ar_  =  the saturation state of aragonite

### Experimental Incubations and Response Variables

Algal samples were allocated randomly to one of three pCO_2_ treatments: 1) ambient pCO_2_ (∼400 µatm), 2) elevated pCO_2_ (∼660 µatm), and 3) a variable pCO_2_ treatment that cycled between 400 and 660 µatm (Figure 1). The elevated pCO_2_ was chosen based on pre-dawn measurements of pCO_2_ at the downstream collection site (Table 2). Prior to placing coralline algal samples into the treatments, each sample was buoyant weighed with the epoxy base (to the nearest mg) [78]. Six samples from each habitat origin (upstream, downstream) were placed on elevated PVC racks in each tank (15 cm above the bottom). The ambient controls and pCO_2_ treatments were fully replicated twice (N = 2 tanks). The variable pCO_2_ treatment was accomplished by placing samples in an ambient pCO_2_ tank for 18 h (from 0600 to 0000) and then transferring the samples to a dedicated elevated pCO_2_ tank (*i.e*., samples in different treatments were not mingled) for 6 h (from 0000 to 0600) each day to simulate the timing of elevated pCO_2_ in downstream habitats in the field (Figure 1B). At each time of transfer, samples from the ambient and elevated treatments also were removed from their tanks and returned to the same tank to serve as a sample handling control.

**Table 2:** Daily integrated pCO_2_ for upstream and downstream backreef habitats.

Habitat	Diel Period	pCO_2_ (µatm)
Upstream	Day	424.8±9.4
	Night	421.0±9.9
Downstream	Day	385.8±5.9
	Night	700.4±55.5

Values (means ± SE) of *in situ* pCO_2_ from daily integrated water samples at upstream and downstream sites of collection (N = 5 days). Upstream sites were located at the reef crest and downstream sites were located 200 m shoreward of the reef crest. Continuous water samples were collected simultaneously and autonomously from each site during the day (from 0800 to 1800) and during the night (from 1900 to 0400).

After 14 days in treatment conditions, all coralline algal samples were buoyant weighed again and 3 samples from each origin and pCO_2_ treatment replicate were chosen randomly for measurements of photosynthesis and respiration. Photosynthesis measurements were estimated during the day from changes in dissolved oxygen during 30-min incubations of each sample in a 102-ml acrylic chamber fitted with a PreSens dipping oxygen optode connected to a Fibox 3 transmitter (Precision Sensing GmbH, Germany). The oxygen probe was calibrated at the start of incubations using a 2-point calibration with temperature correction in water-saturated air (100%) and seawater with no oxygen (supersaturated sodium dithionite, Na_2_S_2_O_4_). Oxygen concentrations automatically were corrected for temperature using a PreSens probe that simultaneously measured temperature in the incubation chamber (Precision Sensing GmbH, Germany). A water jacket surrounding the acrylic chamber was connected to a circulating water bath (Lauda) that provided temperature control (28°C). A stir bar at the base of the chamber created vigorous water motion. Light was provided by two fiber optic halogen lights that created PAR levels in the chamber similar to those in the mesocosms (∼600 µmol quanta m^−2^ s^−1^). Following photosynthesis measurements, samples were returned to their respective treatments and respiration estimates were made on the same samples at night between 1900 and 2300. During respiration measurements the chamber was kept dark using an opaque shroud. For each incubation (photosynthesis and respiration), the chamber was filled completely with seawater from the appropriate treatment and new seawater was used for each incubation. Seawater from the ambient treatment was used for both ambient and variable treatment incubations (because samples in the variable treatment were not placed in the elevated treatment until 0000), and seawater from the elevated treatment was used for the elevated incubations. Oxygen concentrations were recorded each minute and photosynthesis and respiration rates were calculated from the linear slopes over time. Metabolic rates were normalized to surface area of the coralline algal samples, estimated by image analysis (ImageJ, NIH) of digital photographs of each sample. Net calcification rates for each sample were calculated by converting the buoyant weight gained during the experiment to the dry weight gained using the density of calcite (2.71 g cm^−3^) [78] and normalizing the rates to surface area.

### Statistical Analyses

Normalized rates of net calcification, gross photosynthesis, respiration, and net photosynthesis were analyzed using a mixed model ANOVA, with habitat origin (upstream/downstream) and pCO_2_ treatment (ambient, elevated, variable pCO_2_) as fixed factors and tank as a random nested factor. If the tank factor was not significant in the initial analysis (P>0.25), it was eliminated from the subsequent analysis and a two-way fixed factor ANOVA was used [79]. Additionally, an explicit *a priori* contrast tested specifically for the effect of the variable pCO_2_ treatment on coralline algae from upstream and downstream habitats. The assumptions of normality and homoscedasticity were examined prior to all analyses through the examination of residuals and Cochran's test, respectively.

## Results

### Response Variables

**Figure 2:**
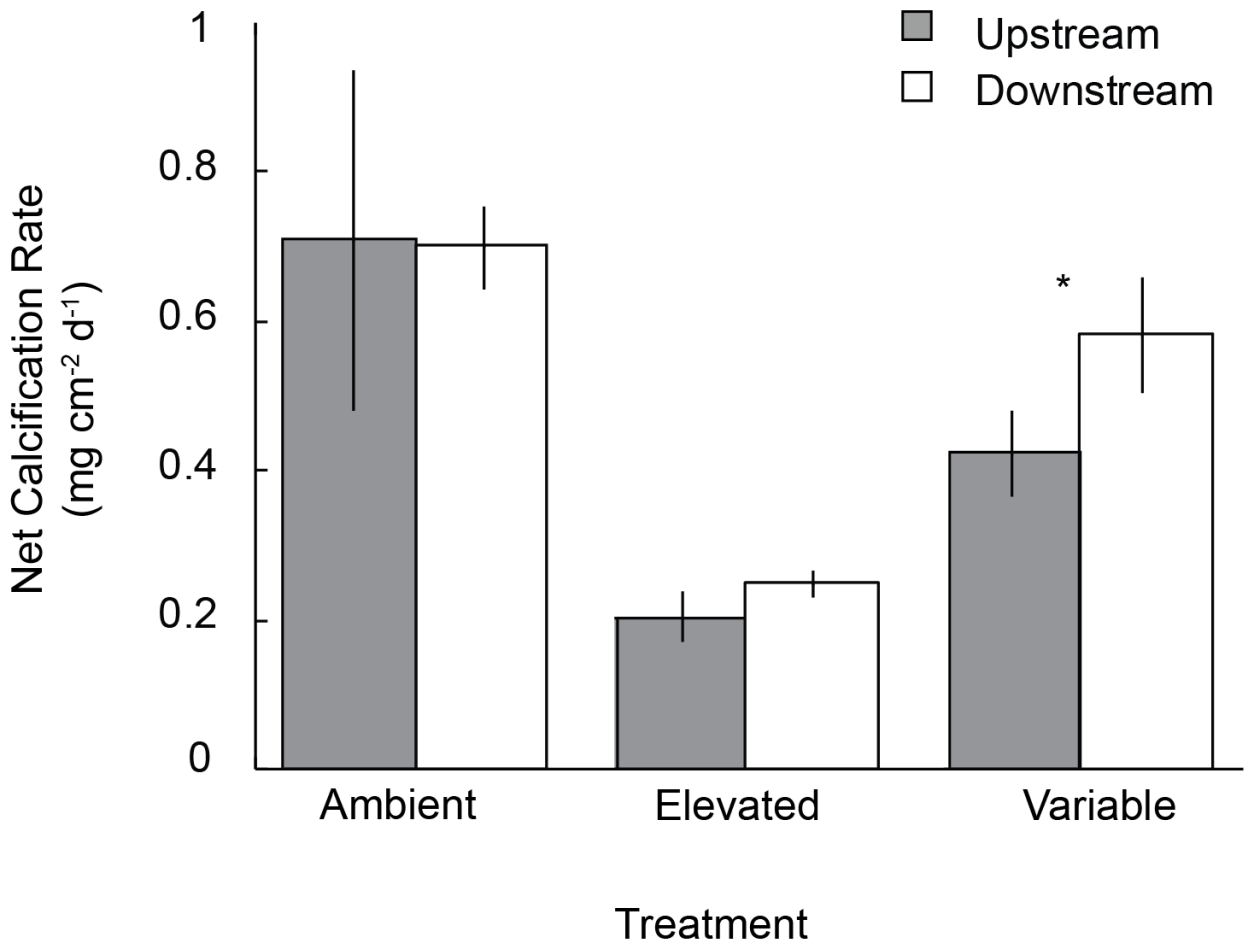
Calcification response of *Porolithon onkodes* to ocean acidification treatments. Mean (±SE) rates of net calcification for *P. onkodes* collected from upstream and downstream habitats after 14 days of exposure to three pCO_2_ treatments. The asterisk denotes a significant difference of an *a priori* comparison between upstream and downstream origins under the variable pCO_2_ treatment.

The nested tank effect in the mesocosm experiment was not significant (P>0.25) in the initial analysis of net calcification so it was removed from the subsequent analysis [79]. Net calcification of *P. onkodes* was significantly affected by an interaction between the pCO_2_ treatment and habitat origin (F_2,42_ = 4.02, P = 0.025), with significant effects of both pCO_2_ treatment (F_2,42_ = 18.42, P<0.0001), and habitat origin (F_1,42_ = 5.18, P = 0.028) (Figure 2, Table S1). As demonstrated in previous studies, net calcification was reduced by over 70% in the elevated pCO_2_ treatment compared to ambient conditions and this trend was exhibited by individuals from both upstream and downstream habitats. The overall response of *P. onkodes* net calcification to the variable pCO_2_ treatment was an intermediate decrease that might be explained most parsimoniously by the proportion of time spent in the ambient (75%) and elevated (25%) pCO_2_ treatments, although the overall decrease in calcification was closer to 35%. Notably, the *a priori* comparison between *P. onkodes* collected from upstream and downstream habitats revealed significantly different calcification responses to the variable pCO_2_ treatment (F_1,14_ = 22.43, P<0.001). Following exposure to the variable pCO_2_ treatment, corallines from the downstream habitats experienced a 17% decrease in net calcification as compared to the ambient controls, while corallines from the upstream habitats experienced a 41% decrease in calcification compared to ambient controls (Figure 2).

**Figure 3:**
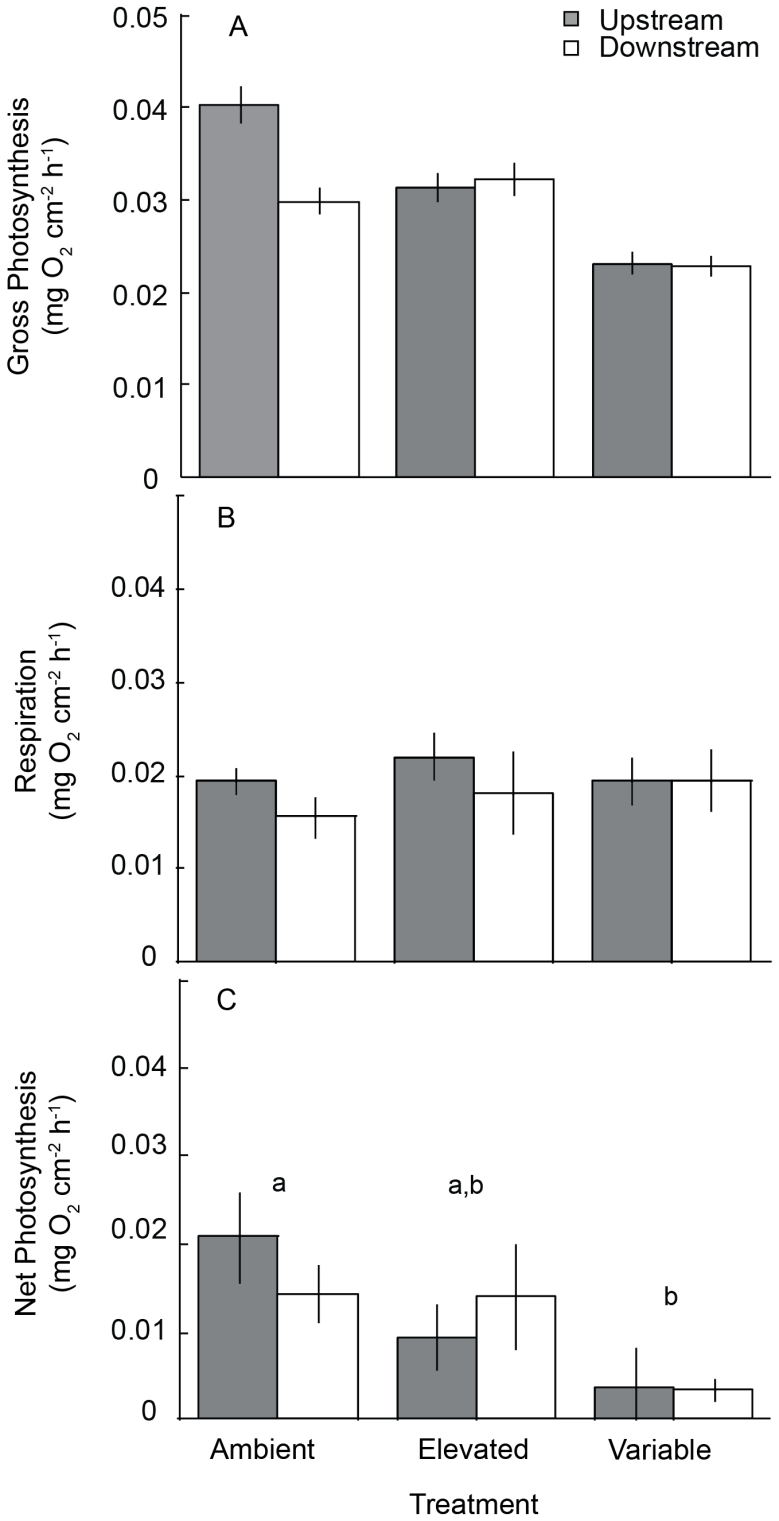
Metabolic responses of *Porolithon onkodes* to ocean acidification treatments. Mean (±SE) rates of gross photosynthesis (A), respiration (B), and net photosynthesis (C) of *P. onkodes* collected from upstream and downstream habitats after 14 days of exposure to three pCO_2_ treatments. There were no significant differences among treatments or origins for gross photosynthesis. Small case letters represent results of *post hoc* multiple comparisons for net photosynthesis where treatments sharing letters are not significantly different.

There was no significant effect of nesting tank within treatment in the initial analyses of the metabolic response variables (P>0.25), therefore the tank effect was pooled in subsequent analyses [79]. Rates of gross photosynthesis were not affected significantly by pCO_2_ treatment (F_2,18_ = 3.03, P = 0.086), habitat origin (F_1,18_ = 0.630, P = 0.443), or the interaction between factors (F_2,18_ = 0.738, P = 0.499) (Fig. 3A, Table S1). Similarly, there was no effect of pCO_2_ treatment (F_2,18_ = 0.414, P = 0.667), habitat origin (F_1,18_ = 1.26, P = 0.276), or the interaction between factors (F_2,18_ = 0.020, P = 0.980) on *P. onkodes* rates of respiration (Fig. 3B, Table S1). However, the net photosynthetic rate of *P. onkodes* was significantly lower in the variable pCO_2_ treatment compared to the ambient treatment (F_2,18_ = 5.59, P = 0.013). There was no effect of habitat origin (F_1,18_ = 0.041, P = 0.843) or the interaction between factors (F_2,18_ = 0.882, P = 0.431) on net photosynthesis (Fig. 3C, Table S1). Data are archived at the Moorea Coral Reef LTER data repository (http://mcr.lternet.edu/data/).

## Discussion

Our results demonstrate that the response of a coralline alga to variability in pCO_2_ depends upon the conditions in the original habitat. *Porolithon onkodes* collected from a downstream habitat, with prior exposure to high pCO_2_ variability, calcified significantly more in the variable pCO_2_ treatment than individuals from the upstream habitat, with history of exposure to lower and diurnally stable pCO_2_. These findings support our hypothesis that exposure to oscillating, high pCO_2_ within the natural range of variability, had a modulating effect on coralline calcification compared to the static high pCO_2_ treatment. We suggest that because the original habitat of *P. onkodes* had a significant impact on the coralline calcification response to variable pCO_2_, a history of exposure to dynamic carbonate conditions may facilitate plasticity in organismal responses to ocean acidification. Our findings emphasize the importance of incorporating oscillating pCO_2_ levels, where appropriate, into laboratory experiments because prior conditioning to high pCO_2_ may confound calcification responses to ocean acidification.

Corallines exposed to the static treatment of high CO_2_ calcified >70% less than those in the ambient treatment, regardless of the origin of collection. This finding corroborates several recent studies that have shown static, high pCO_2_ conditions to have severe consequences for crustose coralline calcification and growth [40], [55]–[60], [80]. The reduction in calcified biomass following exposure to high pCO_2_ may be a result of decreased calcification and/or increased dissolution associated with a lower Ω. Although the high pCO_2_ treatments did not reach undersaturation with respect to aragonite or calcite saturation state (Ω <1), calcification becomes increasingly difficult and potentially energetically more costly as it approaches the saturation horizon (Ω = 1) [3]. The difference in the reduction of net calcification for upstream and downstream organisms between the variable treatment and ambient pCO_2_ treatments (41% and 17%, respectively) indicates that *P. onkodes* from downstream habitats were able to mitigate some of the negative effects of elevated pCO_2_ on calcification, and/or the promoting effects of higher pCO_2_ on calcium carbonate dissolution. The response of a marine calcifier to oscillating pCO_2_ in a laboratory ocean acidification experiment has been demonstrated in few other studies, thus far. Dufault *et al.* (2012) showed that coral recruits exposed to oscillating pCO_2_ (between ∼450 and ∼850 µatm) experienced enhanced growth and survival compared to recruits exposed to both a static ambient and high pCO_2_ treatment [36]. Together these findings indicate that variability in carbonate chemistry should be incorporated into future OA experiments in order to enhance the ecological relevance of laboratory manipulations.

The increase in dissolved CO_2_ associated with ocean acidification and CO_2_ enrichment has the potential to fertilize photosynthesis by increasing the amount of substrate available to the photosynthetic enzyme Ribulose-1,5-bisphosphate carboxylase oxygenase (RuBISCO) [81], [82]. High pCO_2_ has been shown to stimulate photosynthesis in a variety of marine primary producers including seagrasses, phytoplankton, and some macroalgae [39], [81], [82]. However, species-specific responses may be influenced by the presence and activity of carbon concentrating mechanisms (CCMs) and whether a primary producer is carbon limited. We hypothesized that CO_2_ enrichment would stimulate photosynthetic rates in *P. onkodes* by providing more substrate for carbon fixation. We found no effect of pCO_2_ treatment on rates of *P. onkodes* gross photosynthesis or respiration, but a significant effect of the pCO_2_ treatment on net photosynthesis, with highest rates in the ambient pCO_2_ treatment, and lowest rates in the variable pCO_2_ treatment. Because net photosynthesis was determined based on gross photosynthesis and respiration rates, our finding may be a statistical result that has little biological basis. The role of CCMs in coralline algae is not well understood, and differences in CCM activity may have contributed to the mixed results found here and in the recent literature. For example, Anthony et al. (2008) found that exposure to extremely high pCO_2_ conditions (pH 7.6 and 7.8) decreased *P. onkodes* net productivity by 50% to 100%, respectively [55]. Furthermore, Martin et al. (2013) found that high pCO_2_ (700 µatm) decreased net photosynthetic efficiency of the Mediterranean coralline alga *Lithophyllum cabiochae*, and similar to our study found high pCO_2_ to have no effect on coralline respiration [83]. Respiration measurements were conducted between 1900 and 2300 using water from the appropriate treatments. For the variable pCO_2_ treatments, samples were incubated with ambient water because samples were not placed into high pCO_2_ conditions until midnight (0000). This methodology did not account for the response of the variable treatment samples to high pCO_2_ and, therefore, may underestimate the impact of the variable pCO_2_ treatment on rates of coralline respiration.

We suggest that the effect of habitat origin on the *P. onkodes* calcification response to variable pCO_2_ provides evidence of *in situ* acclimatization to naturally occurring variable pCO_2_. Although the terms ‘acclimation’ and ‘acclimatization’ often are used interchangeably, here we follow the terminology of Edmunds and Gates (2008), wherein acclimatization refers to the physiological compensation of an organism to a regime of natural co-occurring environmental stimuli [84]. The relatively short distance between sites (200 m) and lack of physical barriers makes the possibility of genetic differentiation, thus adaptation, between upstream and downstream coralline populations unlikely. If local adaptation were the underlying mechanism to the significance of the origin by pCO_2_ treatment interaction, we would expect both upstream and downstream individuals to exhibit the same response to variable pCO_2_. Form & Reibesell (2012) were among the first to document an example of acclimation of a marine calcifier to acidified conditions [42]. The cold-water coral, *Lophelia pertuse*, acclimated to acidified conditions after six months of exposure in a controlled laboratory mesocosm [42]. This provides an example of temporal acclimation to conditions similar to ocean acidification. In the current study, we suggest that corallines from downstream habitats are acclimatized to high pCO_2_, but only in the context of oscillating conditions that simulated the natural environment.

It is difficult to discern the exact mechanisms driving lower coralline calcification rates in the static and variable pCO_2_ treatments, in part because calcification in coralline algae is not well understood. Corallines calcify intracellularly, where high-Mg calcite is deposited directly within the organic matrix of the cell wall [47], [85]. In order for intracellular CaCO_3_ precipitation to occur, the internal microenvironment must be supersaturated with respect to CaCO_3_ (high pH). Calcification in corallines may be either biologically controlled or biologically induced [85] and influenced by both photosynthesis and calcification because they are inextricably linked. Biological control over calcification may be driven by photosynthesis, where photosynthesis directly provisions the energy necessary for calcification. Calcification may also be biologically induced as photosynthesis indirectly maintains the chemical conditions needed for CaCO_3_ precipitation by producing OH^−^ ions [86]. For example, precipitation of magnesite and dolomite in *P. onkodes* cells is thought to be biologically induced by changes in pH due to metabolic activity [85]. Calcification is also influenced by external seawater chemistry, because calcified algae must uptake calcium ions (Ca^2+^) and carbonate ions (CO_3_^2−^) directly from the seawater or through the intracellular conversion of HCO_3_^−^ to CO_3_^2−^ [87], [88]. Biomineralization becomes more difficult when the CaCO_3_ saturation state (Ω) approaches undersaturation (<1) [3], as is predicted to occur in the near future with ocean acidification.

Plasticity in the coralline response to high pCO_2_ variability therefore may have been driven by mechanisms associated with both photosynthesis and calcification. Identifying the mechanisms that promote coralline acclimatization to variable pCO_2_ was beyond the scope of this study. Here we propose potential biological and mineralogical mechanisms that may have contributed to the differences in coralline calcification by habitat. Two mechanisms (assuming biological control over calcification) by which corallines may maintain intracellular pH at the site of calcification, thus facilitating acclimatization to variable pCO_2_, are: 1) enhancement of photosynthesis from increased dissolved CO_2_ concentrations, and 2) opportunistic use of HCO_3_− to mitigate the decrease in CO_3_^2−^. Photosynthesis may be fertilized by high CO_2_ and thus facilitate supersaturation of the intracellular microenvironment. However, since photosynthesis was not higher in the variable pCO_2_ treatment, this mechanism likely does not explain our calcification results. The second proposed mechanism, bicarbonate usage, applies to algae such as crustose corallines that can utilize HCO_3_^−^ [88] through the conversion of HCO_3_^−^ to CO_2_ with the enzyme carbonic anhydrase. The enzyme-catalyzed conversion releases a hydroxyl ion (OH^−^) and increases the local pH environment in favor of CaCO_3_ precipitation [54]. CCMs, including carbonic anhydrases (CA) and HCO_3_^−^ transporters, are important for supplying carbon to photosynthesis and calcification [89], [90]. Production and expression of CCMs is energetically costly and are influenced by environmental conditions [90], [91]. The temperate coralline alga, *Corallina officinalis*, exhibited a trend of increasing internal CA activity following exposure to high pCO_2_ [92], and an inverse trend between external CA activity and CO_2_ concentration [93]. Corallines frequently exposed to high pCO_2_ therefore may regulate CCMs, such as CA activity, in order to mitigate decreased CO_3_^2−^ concentrations associated with OA by switching to HCO_3_^−^ utilization [88]. A third mechanism (assuming abiotic control over calcification) may have been differences in calcification due to mineralogy. Coralline crusts from different habitats may exhibit differences in magnesium content, and thus corallines from upstream and downstream habitats may have differed in mineralogy from the start of the experiment. Mineralogy, rather than acclimatization, may have driven the calcification response to variable pCO_2_ if abiotic calcification (cell infilling) by magnesite and dolomite [85] was affected differentially by pCO_2_ conditions. In order to conclusively differentiate between mineralogical and physiological mechanisms, future studies should quantify coralline mineralogy before and after exposure to treatment conditions.

Based on our findings, biogenic calcifiers may be found in greater abundances at microhabitats with lower daily pCO_2_ variability, a finding that corroborates field-based studies that showed the distribution and abundance of benthic calcifiers to be correlated with pCO_2_/pH variability [12]–[14], [16]. As seen in other studies of shallow reef flats [7], [12], our data show that shallow reef environments may be shaped by diel pH variability. Our findings are corroborated by seawater chemistry measurements from a shallow reef system in Okinawa, Japan, where the alkalinity and DIC are consistently lower on the back reef during the day than on the reef crest [10]. Furthermore, the Tiahura reef transect, a similar section of back reef 6.5 km west of our sites on the north shore of Moorea, has been revisited since the early 1980's and calcification and carbonate chemistry have been thoroughly characterized, supporting our characterization of the upstream and downstream sites as having high and low pCO_2_ variability respectively [31], [67], [94]–[97]. In addition to differences in the magnitude of carbonate chemistry variability, other factors that may influence coralline growth rates within a habitat include grazing intensity, nutrient availability, water flow, and light levels. Our coralline samples were collected from the same depth at upstream and downstream sites and samples were acclimated to the same light conditions prior to the start of the experiment. Previous light history in the original habitat (at the same depths) likely did not impact the observed growth rates among treatments. Although upstream and downstream habitats may be characterized by different flow regimes, this experiment was conducted during the Austral winter (May-June), when differences in flow between the upstream and downstream site are minimal [66]. Furthermore, flow-through water within mesocosm tanks, aeration, and water pumped from the chilling system created water flow within the tanks (∼10 cm s^−1^). Although many factors influence coralline growth, the significant results from our laboratory experiment and the previous literature suggest that changes in the patterns of calcification rate among treatments were driven most likely by the carbonate chemistry conditions in the original habitats.

This study documents a modulated response of *P. onkodes* calcification to high pCO_2_ in the oscillating OA treatment which was based upon the natural range of pCO_2_ experienced *in situ*. The significant impact of the original habitat on the coralline calcification response to pCO_2_ treatments indicates that variability in carbonate chemistry likely is an important factor influencing biogenic calcification rates. These results are important because they emphasize that the original environmental conditions of individuals used in OA experiments can have a significant effect on experimental outcomes. Variability in carbonate chemistry in the original habitat may be a potential confounding factor in past OA studies, and may explain some of the ambiguity in calcification results in the literature. Further, we suggest that organisms in highly variable pCO_2_ environments may be acclimatized to near-future changes associated with ocean acidification. Studies should continue to explore the adaptive potential of marine organisms to the changing environment by exploring both *in situ* acclimatization and adaptation. Our findings have important implications applicable to other ecosystems that experience similar diel fluctuations in pH such as upwelling regions and estuarine systems. Given the potential dire consequences of ocean acidification for coastal ecosystems it is important to consider how acclimatization may facilitate the survival of marine organisms in the near future.

## Supporting Information

Table S1 **ANOVA tables.** Results from two-way ANOVA, with pCO_2_ treatment and habitat origin as fixed and interacting factors. Each response variable was analyzed separately. During initial analyses tank was treated as a random factor nested within treatment, but was not significant for all response variables (P>0.25) and was removed from subsequent analyses. Significant values (P<0.05) are highlighted in bold.
(DOCX)
