# Supplementary material for: Acclimatization of the Crustose Coralline Alga Porolithon onkodes to Variable pCO2
Source: PLoS One. 2014 Feb 5;9(2):e87678. doi: 10.1371/journal.pone.0087678 (PMC3914853; doi:10.1371/journal.pone.0087678)
Supplement: Table S1 — ANOVA tables. Results from two-way ANOVA, with pCO2 treatment and habitat origin as fixed and interacting factors. Each response variable was analyzed separately. During initial analyses tank was treated as a random factor nested within treatment, but was not significant for all response variables (P>0.25) and was removed from subsequent analyses. Significant values (P<0.05) are highlighted in bold. (DOCX) [file pone.0087678.s001.docx]

**Supporting Information**

**Table S1. ANOVA tables.** Results from two-way ANOVA, with pCO_2_ treatment and habitat origin as fixed and interacting factors. Each response variable was analyzed separately. During initial analyses tank was treated as a random factor nested within treatment, but was not significant for all response variables (P>0.25) and was removed from subsequent analyses. Significant values (P<0.05) are highlighted in bold.

| **Source** | **SS** | **DF** | **MS** | **F** | **P** |
| --- | --- | --- | --- | --- | --- |
| *Calcification* |  |  |  |  |  |
| pCO_2_ treatment | 1.833 | 2 | 0.916 | 18.42 | **<0.0001** |
| habitat origin | 0.258 | 1 | 0.258 | 5.176 | **0.028** |
| pCO_2_ x habitat | 0.400 | 2 | 0.200 | 4.019 | **0.025** |
| Error | 2.090 | 42 | 0.050 |  |  |
| *Gross Photosynthesis* |  |  |  |  |  |
| pCO_2_ treatment | 0.00062 | 2 | 0.00031 | 3.028 | 0.086 |
| habitat origin | 0.00006 | 1 | 0.00006 | 0.629 | 0.443 |
| pCO_2_ x habitat | 0.00015 | 2 | 0.00008 | 0.738 | 0.499 |
| Error | 0.0020 | 18 | 0.00001 |  |  |
| *Respiration* |  |  |  |  |  |
| pCO_2_ treatment | 0.00005 | 2 | 0.00002 | 0.414 | 0.667 |
| habitat origin | 0.00007 | 1 | 0.00007 | 1.263 | 0.276 |
| pCO_2_ x habitat | 0.00000 | 2 | 0.00000 | 0.020 | 0.980 |
| Error | 0.0090 | 18 | 0.00005 |  |  |
| *Net Photosynthesis* |  |  |  |  |  |
| pCO_2_ treatment | 0.00079 | 2 | 0.00040 | 5.588 | **0.013** |
| habitat origin | 0.00000 | 1 | 0.00000 | 0.041 | 0.843 |
| pCO_2_ x habitat | 0.00013 | 2 | 0.00006 | 0.882 | 0.431 |
| Error | 0.00128 | 18 | 0.00007 |  |  |
